# Bone Marrow Mesenchymal Stem Cells Attenuate Mitochondria Damage Induced by Hypoxia in Mouse Trophoblasts

**DOI:** 10.1371/journal.pone.0153729

**Published:** 2016-04-21

**Authors:** Lingjuan Wang, Xiaoyan Xu, Lina Kang, Wenpei Xiang

**Affiliations:** 1 Family Planning Research Institute, Tongji Medical College, Huazhong University of Science and Technology, Wuhan, China; 2 Department of Obstetrics and Gynecology, Tongji Hospital, Tongji Medical College, Huazhong University of Science and Technology, Wuhan, China; East Carolina University, UNITED STATES

## Abstract

**Objective:**

We aimed to observe the change of mitochondrial function and structure as well as the cell function induced by hypoxia in mouse trophoblasts, and moreover, to validate the restoration of these changes after co-culture with bone marrow mesenchymal stem cells (hereinafter referred to as “MSCs”). Further, we explored the mechanism of MSCs attenuating the functional damage of trophoblasts caused by hypoxia.

**Methods:**

Cells were divided into two groups, trophoblasts and MSCs+trophoblasts respectively, and the two groups of cells were incubated with normoxia or hypoxia. Chemiluminescence was used to assay the β-HCG and progesterone in cell culture supernatants quantitatively. Western blotting and PCR were applied to detect the expression of Mfn2, MMP-2, MMP-9 and integrin β1 in the two groups. The mitochondrial membrane potential of each group of cells was detected with JC-1 dye and the ATP content was measured by the phosphomolybdic acid colorimetric method. We utilized transmission electron microscopy for observing the ultrastructure of mitochondria in trophoblasts. Finally, we assessed the cell apoptosis with flow cytometry (FCM) and analyzed the expression of the apoptosis related genes—*Bcl-2*, *Bax*, *Caspase3* and *Caspase9* by western blotting.

**Results:**

The results showed that the Mfn2 expression was reduced after 4 h in hypoxia compared with that in normoxia, but increased in the co-culture group when compared with that in the separated-culture group (*p*<0.05). In addition, compared with the separated-culture group, theβ-HCG and progesterone levels in the co-culture group were significantly enhanced (*p*<0.05), and so were the expressions of MMP-2, MMP-9 and integrin β1 (*p*<0.05). Moreover, it exhibited significantly higher in ATP levels and intensified about the mitochondrial membrane potential in the co-culture group. TEM revealed disorders of the mitochondrial cristae and presented short rod-like structure and spheroids in hypoxia, however, in the co-culture group, the mitochondrial cristae had a relatively regular arrangement and the mitochondrial ultrastructure showed hyperfusion. The expression of Bax, Caspase3 and Caspase9 was decreased in the co-culture group when compared with that in trophoblast cells cultured alone (*p*<0.05), while the Bcl-2 levels and the Apoptosis Index (AI) were markedly increased in the co-culture group (*p*<0.05).

**Conclusion:**

Bone marrow mesenchymal stem cells can attenuate mitochondria damage and cell apoptosis induced by hypoxia; the mechanism could be upregulating the expression of *Mfn2* in mouse trophoblasts and changing mitochondrial structure.

## Introduction

Trophoblast cells were crucial to the progress of embryo implantation and were indispensable for nourishing placental development [1,2]. Especially, the extrovillous trophoblast can accomplished the process of placental artery remodeling via differentiation, migration and invasion in uterine wall and it was the key process to maintaining maternal-fetal communication [1,3,4]. However, the dysfunction of trophoblast caused by chronic hypoxia, which was associated with preeclampsia or other pathogenies, may result in incomplete vascular remodeling and limits to maternal-fetal communication, and could even lead to fetal intrauterine growth restriction [5–7].

Many dates suggested that the cells, when exposed to hypoxia, would lead to inadequate oxygen supply and metabolism in disorder, and then even undergo apoptosis—an initiative cell death process with caspase-3 activation [8, 9]. The major signal transduction pathways about apoptosis acted through the external death receptor pathway, intrinsic mitochondrial pathways and endoplasmic reticulum stress pathways [10, 11], among which the endogenous apoptosis pathway mediated by mitochondria was the main route of programmed cell death in mammals [12, 13]. It would appear to swell and increase permeability for the mitochondrial membrane after stimulated by apoptosis-related factors, such as hypoxia, metabolic disturbance of energy-ATP, DNA damage, toxic effect, increased ROS, cell cycle arrest and so on, and then apoptosis-related active substances including cytochrome C, endonuclease of apoptosis inducing factor and Bcl-2 inhibitor of transcription 1 (Bit1) originally located in the mitochondria were released and than activated regulatory proteins of caspase cascade as well as the members of Bcl-2 family to mediate apoptosis [14–18]. Additionally, the death receptor pathway may also be of relevance with mitochondrial pathway, since the central converging points of these pathways are mitochondria [11,19], and so was the endoplasmic reticulum pathway although it remained to be further explored for the mechanism of cell apoptosis [20–22].

MSCs belong to a kind of pluripotent stem cells, which represent a new promixing approach for repairing damaged tissue and the frontier of regenerative medicine [23]. Several investigations have reported that it could not only ameliorate ovarian structure and function damaged by chemotherapy and restore long-term fertility after transplantation via the caudal vein in mice [24], but it could also reduce the apoptosis of granulosa cells by co-culture [25, 26]. Indeed, a present study has indicated that MSCs had the potential of anti-apoptosis according to the co-culture system of MSCs and targeted cells [27, 28]. Moreover, the exosomes extracted from the MSCs or intercellular transfer of cytoplasmic materials may also attenuated the targeted cells apoptosis and improved mitochondrial membrane potential under hypoxia condition, which contributed to increase cell survival of therapeutic in vitro or transplantation in vivo [29, 30]. Our study aims to observe the change of mitochondria and cell apoptosis induced by hypoxia in trophoblasts and demonstrates that MSCs can successfully attenuate cell damage, and further explore the mechanism of MSCs restoring the function in mouse trophoblasts.

## Material and Methods

### Materials

Eagle’s Minimum Essential Medium (EMEM), Dulbecco's Modified Eagle Medium (DMEM), penicillin and streptomycin were purchased from Invitrogen (Grand Island, NY). Fetal bovine serum and calf serum were purchased from Hyclone Laboratories (Green Bay, WI). All other chemicals were from Sigma Chemical Co. (St. Louis, MO).

### MSCs and Trophoblasts Isolation and Culture

Following approval from the Animal Research Center of Huazhong University of Science and Technology, primary MSC were isolated and purified from C57BL/6 (WT) mice beween 8 and 12 weeks of age, from which the femurs and tibias were removed out by aseptic operation, washed twice in DMEM. The bone marrow was rushed out of with complete medium (DMEM containing 10% calf serum, 100-units/ml penicillin, 100μg/ml streptomycin, 2mM L-glutamine, and 1mM sodium pyruvate) and centrifuged at 1000g for 5 minutes, than resuspended in complete medium and plated in T25 flasks. Finally, the BMSCs maintained at 37°C in a humidified atmosphere with 5% CO_2_. After 48h, nonadherent cells were removed and replaced with fresh medium. Adherent cells in passage 3 were characterized by flow cytometry for the existence of typical surface markers CD73, CD105, and CD90, as well as the absence of the hematopoietic markers CD34 and CD45. MSCs from passages 3–9 were used in co-culture with trophoblasts.

Trophoblasts were isolated and purified from C57BL/6 (WT) mice and cultured as described. Trophoblasts were suspended in EMEM containing 10% fetal bovine serum, 100-units/ml penicillin, and 100μg/ml streptomycin and then plated at a density of 0.5×10^6^ cells/well in 6-well plates. After overnight culture, the medium was removed and fresh medium was added (containing 5% Fetal bovine serums). Transwell experiments were performed in 6-well transwell plates (0.4μm pore size, BD Biosciences). Trophoblasts (5×10^5^ cells per well) were seeded in the lower chamber and MSCs (5×10^4^ cells per well) were added in the upper chamber 24h later. Hypoxia was achieved by culturing the cells in a tri-gas incubator (Heal Force, Shanghai, China) saturated with 5% CO_2_ and 1% O_2_ at 37°C for the indicated time periods.

### Western Blotting Analysis

Proteins (30μg) were separated by SDS-PAGE and transferred to nitrocellulose membranes. Nonspecific binding was blocked in 5% nonfat milk in Tris-buffered saline (TBS) + 0.1% Tween-20 for 1 h. The membranes were probed overnight with primary antibodies for rabbit anti-Mfn2 (Abcam, Cambridge, MA), rabbit polyclonal anti-Bcl-2 antibody (Cell Signaling, USA), rabbit polyclonal anti-Bax antibody (Cell Signaling, USA), rabbit polyclonal anti-caspase3 antibody (Cell Signaling, USA), rabbit polyclonal anti-caspase9 antibody (Cell Signaling, USA), rabbit polyclonal anti-MMP-2 antibody (Cell Signaling, USA), rabbit polyclonal anti-MMP-9 antibody (Cell Signaling, USA), rabbit polyclonal anti- integrin β1 antibody (Cell Signaling, USA) and rabbit polyclonal anti-β-actin antibody (Santa Cruz Biotech, CA) in TBS-T containing 1% nonfat milk at 4°C, washed three times with TBS-T for 10 min, and were probed with horseradish peroxidase-conjugated secondary antibodies for 1 h. The membranes were washed three times and exposed in a standard enhanced chemiluminescence reaction according to manufacturer’s instructions (Pierce, Rockford, IL.). The results were normalized to β-actin signal intensity.

### RNA isolation, reverse transcription, and polymerase chain reaction (RT-PCR)

Total RNA was extracted from trophoblasts using RNeasy mini extraction kits from Qiagen (Valencia, CA) according to the manufacturer's protocol. cDNA was synthesized using 1μg RNA and oligo dT primers (Qiagen) and Omniscript^™^ reverse transcriptase (Qiagen). PCR reaction mixtures were prepared using SYBR Green PCR master mix (PE Applied Biosystems, Foster City, CA). SYBR Green two-step real-time RT-PCR was performed using forward and reverse primer pairs prevalidated and specific for iNOS, β-actin and PKG (Qiagen). All samples were run in triplicate. The level of gene expression for each sample was normalized to β-actin mRNA expression using the comparative Ct method.

### Measurement of mitochondrial membrane potential (ΔΨm)

Mitochondrial stability was determined by fluorescence microscopy after incubation with JC-1 (5,59,6,69-tetrachloro-1,19,3,39-tetraethylben zimidazolylcarbocyanine iodide; Molecular Probes, Eugene, OR). Trophoblasts were incubated with 0.1μM JC-1 (Cayman Chemical Company, USA) fluorescence reagent for 30min in the CO_2_ incubator and washed with PBS three times. Mitochondrial membrane potential was observed under a fluorescence microscope. The green JC-1 signals appeared at 485/535nm, and the red signals appeared at 590/610nm.

### Detecting ATP levels

To evaluate the mitochondrial function, the ATP levels of trophoblasts were determined. The trophoblasts were suspended in calcium and magnesium ion-free PBS. The reagent (The Shenyang Branch Liang horse Biological Engineering Co, Ltd, China) was dissolved in an ice bath, and the ATP standard solution was diluted to a suitable concentration gradient with double distilled water. 100μl of ATP detection working solution was added to each well of a 96-well plate, and then the solution was blended with pipettor followed by adding 20μl of ATP Standard solution after standing at room temperature for 5 minutes. A standard curve was plotted according to the fluorescence emission values detected with multifunctional microplate reader. Finally, the ATP detection of samples was carried out according to the manufacturer’s instructions and measured the luminescence by automatic microplate reader (BIO-TEK).

### Flow Cytometric Assay for Apoptosis

The trophoblasts were harvested for observation apoptosis. Cells were washed three times with cold PBS and resuspended in 200 μl binding buffer that contained 10μl of annexin V-FITC (R&D) and 5μl propidium iodide (PI) (Sigma, St Louis, MO, United States) for 15 minutes at room temperature while avoiding any light exposure. After incubation, the cells were analyzed with a flow cytometer of EPICS XL Coulter (Beckman-Coulter, United States). All assays were repeated three times.

### Transmission electron microscopy (TEM)

The trophoblasts plated on coverslips were treated as described and then fixed in 2.5% glutaraldehyde overnight, washed with 0.1 M phosphate-buffered saline (PBS, pH 7.2) and fixed in 2% osmium tetroxide for 2 h. The sections were then washed twice with distilled water and stained with 1% uranyl acetate for 1 min and air-dried. Samples were visualized under a JEM-1230 transmission electron microscope (Jeol, Japan).

### Chemiluminescence (CL)

The supernatant of the trophoblasts was harvested and depleted from residual cells by centrifugation, and stored at − 80°C until the chemiluminescence assay was performed. Quantification of β-HCG and progesterone in cell culture supernatants was determined using Chemiluminescence Quantitative Immunoassay Kits (Beckman Coulter, Inc.). Each well of microplate was added with 100μl enzyme conjugate followed by adding 20μl calibrator, control material or samples, then incubated at 37°C for 1h on the oscillators, dried with patting gently after pouring out and washing five times. Finally, 100μl luminous substrate was added into the well of microplate and then measured with a fully automatic Immulite 2000 analyzer (Siemens Healthcare Diagnostic).

### Statistical analysis

Statistical analysis was performed using Statistical Program for Social Science (SPSS Inc., Chicago, IL, USA) software. Each experiment was performed at least three times, and all data are expressed as mean±S.D. Differences between groups were examined by Student’s t-test or multivariate analyses. Values of *p*<0.05 were considered statistically significant.

## Results

### MSCs overcame hypoxia-induced Mfn2 decline in trophoblasts

**Fig 1:**
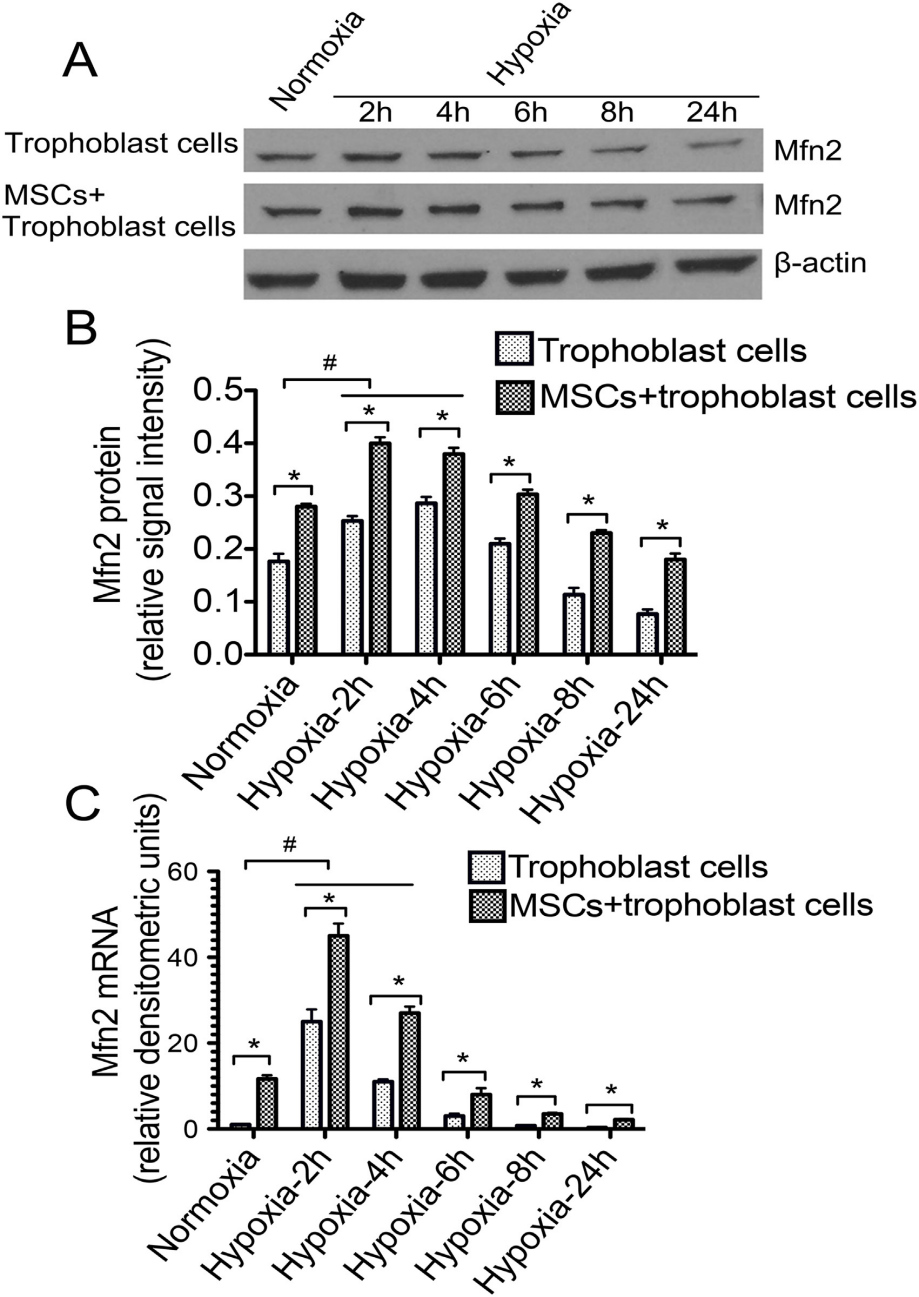
Mfn2 levels in two groups under the condition of normoxia and hypoxia. **A and B**: The most representative image of western blotting for the protein expression level of Mfn2 and the relative signal intensity of Mfn2 protein levels in separate-culture group (trophoblasts) and co-culture group (MSC+trophoblasts). **C**: The comparison of Mfn2 mRNA levels in the two groups and the expression levels by the time in hypoxia. *p<0.05 vs. the corresponding control group.

We measured Mfn2 expression by western blotting and Q-PCR and found, in cells incubated with hypoxia, Mfn2 protein and mRNA levels were increased and may reach the maximal levels between 2 and 4 h (2.2±0.8 fold)(Fig 1). After 4 h, Mfn2 levels started to decline and remained lowest levels at 24 h point. However, Mfn2 protein levels appeared higher in co-culture cells compared to trophoblasts culture alone (Fig 1A and 1B). Q-PCR confirmed these same findings (Fig 1C).

### MSCs improved the function of trophoblasts

**Fig 2:**
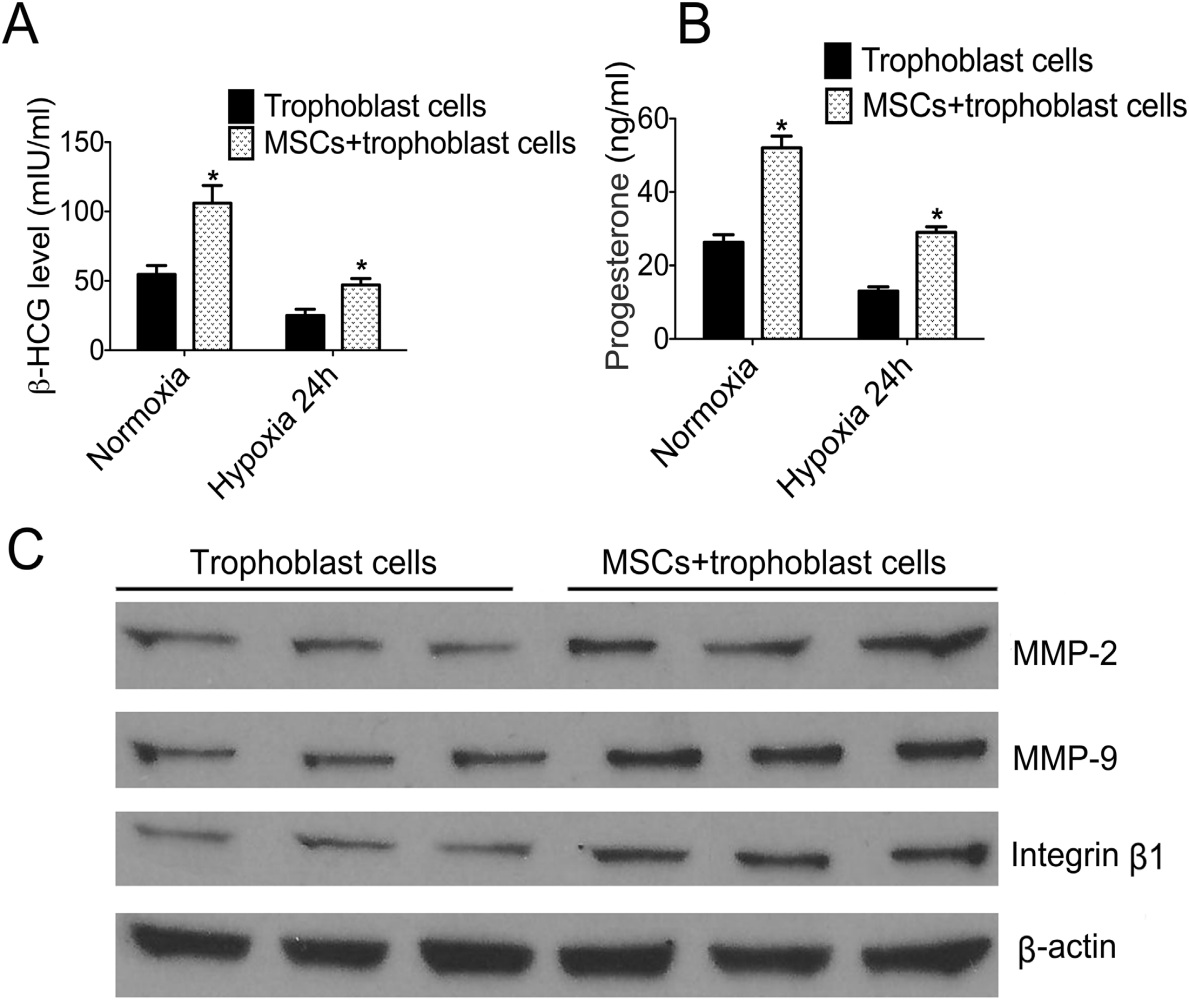
Expressions of hormone levels and function-related proteins of trophoblast cells in two groups. **A**: The comparison ofβ-HCG levels of two groups in normoxia and hypoxia. **B**: The comparison of progesterone levels of two groups in normoxia and hypoxia. **C**: The most representative images of western blotting for MMP-2, MMP-9 and integrin β1 in two groups under the condition of hypoxia. *p<0.05 vs. the corresponding control group.

To test the function including secretory, invasion and adhesion of trophoblast cells, we determined the β-HCG and progesterone levels by chemiluminescence and the expression of MMP-2, MMP-9 and integrin β1 via western blotting. Results showed thatβ-HCG levels have a significant decline in hypoxia cells when compared with those in normoxia. However, β-HCG and progesterone levels were markedly elevated in co-culture cells when compared with those in separate-culture cells (Fig 2A and 2B). In addition, the results indicated that the MMP-2, MMP-9 and integrin β1 levels were significantly increased in the co-culture group when compared with those in the separate-culture group (Fig 2C) when cells incubated with hypoxia. All findings above proved that MSCs improved the trophoblast cells function.

### MSCs attenuated mitochondrial damage induced by hypoxia in trophoblasts

**Fig 3:**
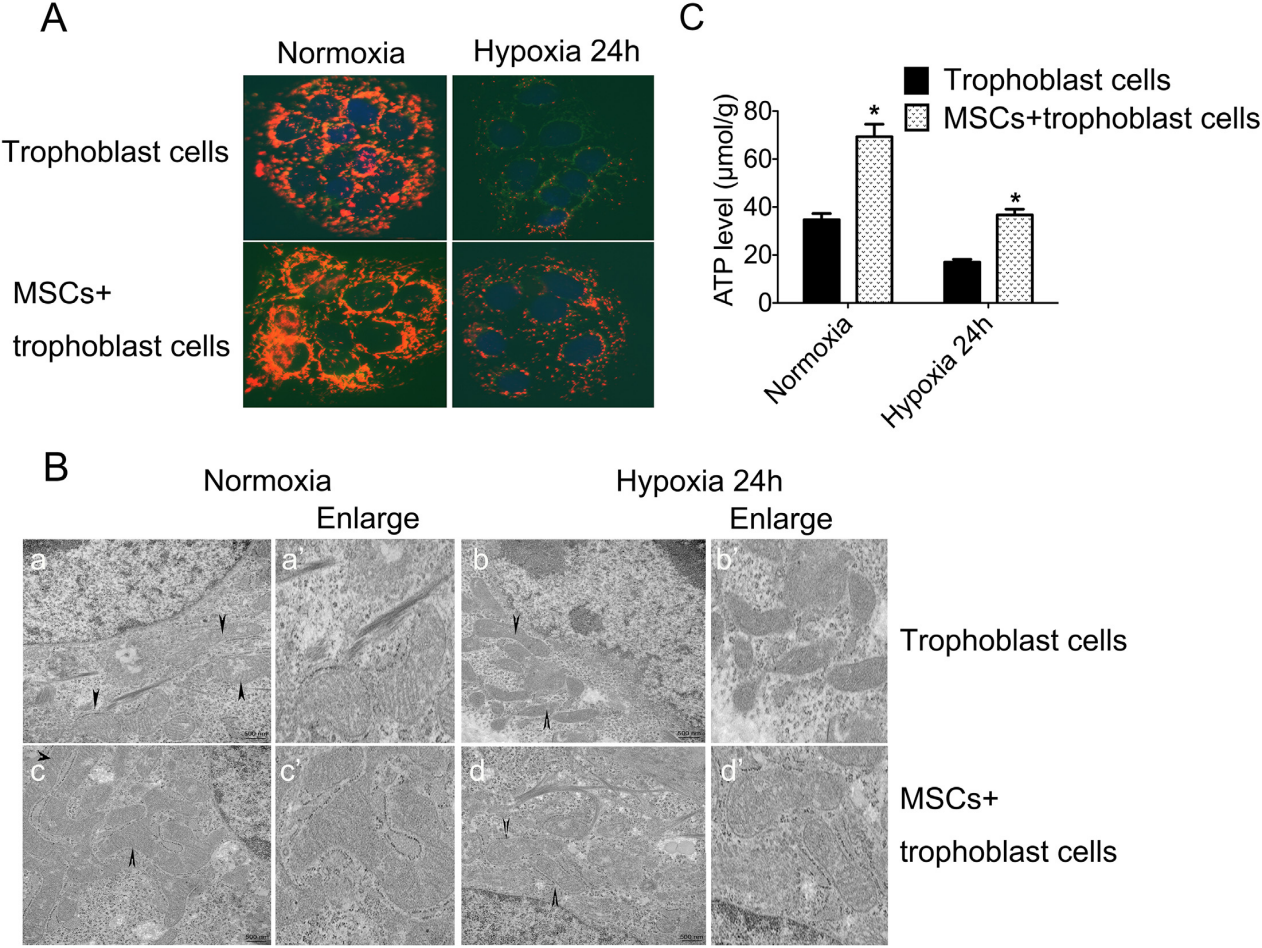
The change of mitochondrial structure and function of two groups in normoxia and hypoxia. **A**: The mitochondrial membrane potential (ΔΨm) was tested by using JC-1; **B**: Mitochondrial ultramicrostructrue were detected by TEM [Ba~Bd: the most representative image of mitochondrial morphology and cristae of trophoblast cells cultured alone or co-cultured in normoxia and hypoxia (black arrow); Ba’~Bd’: higher magnification.] **C**: ATP levels of the two groups in normoxia and hypoxia; *p<0.05 vs. the corresponding control group.

In the two groups, several testing technologies were used to detect mitochondrial function of trophoblast cells in normoxia and hypoxia in order to demonstrate the restoration of mitochondrial damage induced by hypoxia in trophoblasts when co-cultured with MSCs. We measured the mitochondrial membrane potential (ΔΨm) used to assess mitochondrial function with JC-1, and found that a significant attenuated fluorescence intensity was found in trophoblast cells of hypoxia (Fig 3A, Right) when compared with those in normoxia (Fig 3A, Left). In the co-culture group (MSCc + trophoblasts), we found an obvious fluorescence enhancement (Fig 3A, Lower). At the same time, the ATP content was also detected and displayed a marked decrease in hypoxia, but an obvious enhancing and improvement in the co-culture group, particularly when compared to the separated-culture group (Fig 3C).

Transmission electron microscopy (TEM) was used to detect the mitochondrial ultramicrostructure in trophoblast cells, and we found a marked difference in mitochondrial morphologies between two groups. In normoxia, the mitochondria were basically in a state of fusion and the mitochondrial cristae had a relatively regular arrangement (Fig 3B, Left). However, in hypoxia, most of the mitochondria presented short rod-like structure and spheroids, especially the mitochondrial cristae were arranged in disorder (Fig 3B, Right). Nevertheless, in the co-culture group involved in MSCs, we could observe orderly mitochondrial cristae and reduced short rod-like structure and spheroids (Fig 3B, lower).

### MSCs alleviated apoptosis induced by hypoxia in trophoblasts

**Fig 4:**
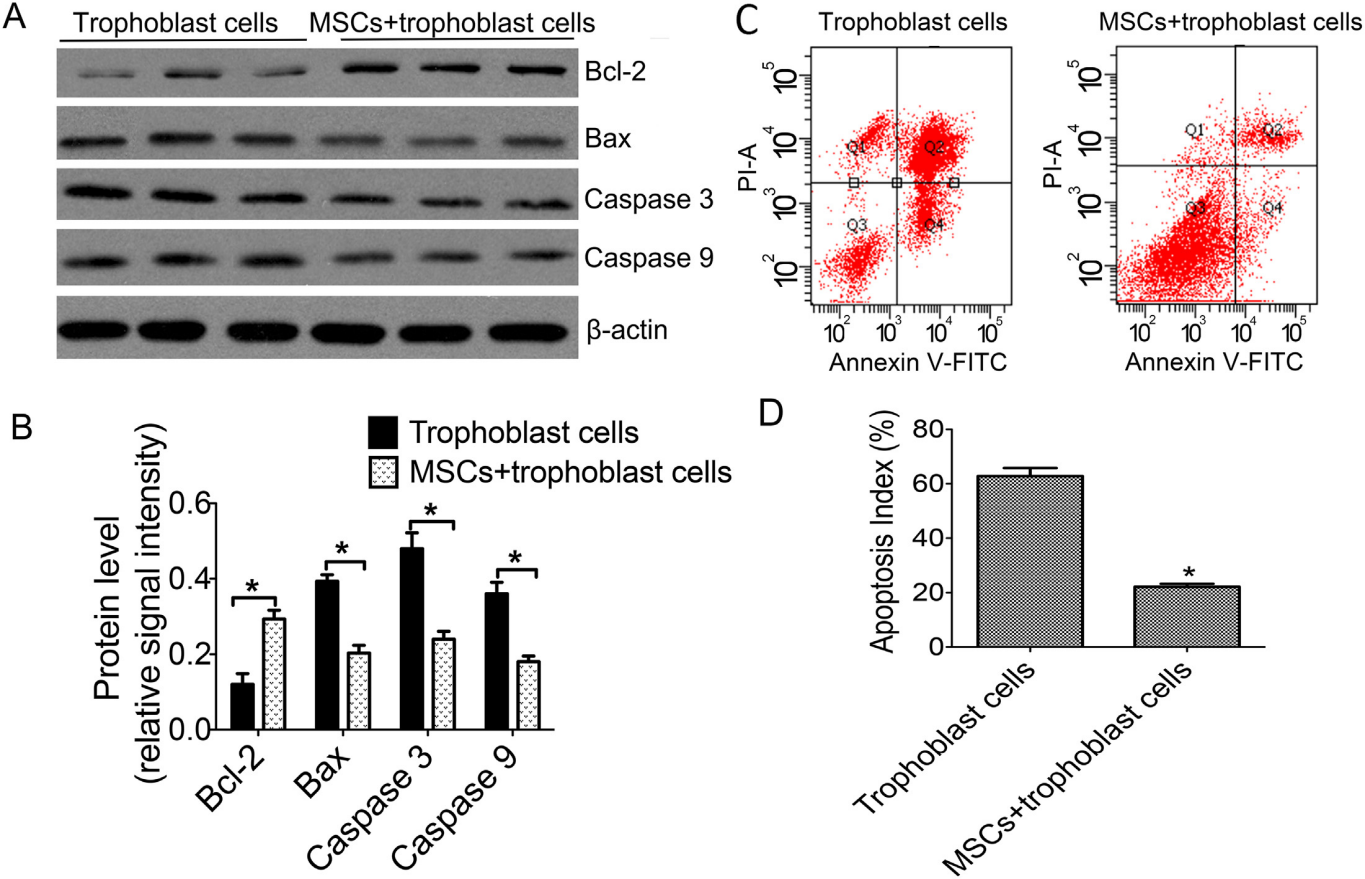
The apoptosis index were detected and the expression of apoptosis-related factors in two groups when incubated with hypoxia. **A**: The most representative images of western blotting for Bcl-2, Bax, caspase 3 and caspase 9 in two groups; **B**: Comparison between Bcl-2 and Bax, caspase 3, caspase 9 protein levels; C: The most representative images of apoptosis in two groups; D The change of Apoptosis Index (AI) in two groups *p<0.05 vs. the corresponding control group.

In hypoxia, flow cytometric assay and western blotting was used to analyze the cells apoptosis and the expressions of anti-apoptotic protein Bcl-2, apoptosis-promoting protein Bax and apoptosis-related factors caspase 3 and caspase 9. Western blotting indicated that the Bax levels were significantly reduced in the co-culture group when compared with trophoblast cells cultured alone and the levels of caspase 3 and caspase 9 remained the same results (Fig 4A and 4B). However, Bcl-2 expression levels were increased in the co-culture group compared to the separated-culture group (Fig 4A and 4B). In addition, the Apoptosis Index (AI) was 22.33% in the co-culture group compared with 62.67% in the separated-culture group, which showed a lower cell apoptosis when involved in MSCs (Fig 4C and 4D). The study revealed that MSCs alleviated apoptosis induced by hypoxia in trophoblasts.

## Discussion

Successful embryo implantation and development depends on the constant process of invasion and adhesion of trophoblast cells, during the course of which placental artery remodeling can be better established to support maternal-fetal communication [2–4]. The secreted hormones derived from trophoblasts are also in need of normal pregnancy maintenance and fetus growth [1]. On the contrary, the reduced or absent invasion and adhesion, which may be associated with hypoxia in the uterine wall, was reported to be at the risk of miscarriage or recurrent spontaneous abortion, intrauterine growth retardation and preeclampsia, and even a leading cause of maternal death and premature birth [31–33].

As we know, trophoblast cells are a major composition of placenta, and the hormones derived from the cells is indispensable in the growth process of the embryo or fetus and is essential for sustaining placental development. In addition, matrix metalloproteinase-2 (MMP-2) and matrix metalloproteinase-9 (MMP-9) mainly located in endochylema of villous trophoblast and syncytiotrophoblast as well as integrin β1 also play an important role in the process of regulating invasion and adhesion in embryonic development [34,35]. In this study, we found that the function of trophoblast cells could be damaged by hypoxia, when co-cultured with MSCs, the levels of hormones, even the function-related protein, were all significantly increased; this suggests that MSCs can improve the function of trophoblasts.

Apoptosis is a highly regulated and initiative cell death process involved in senility, development and injury. The dysregulation of apoptosis will lead to dysfunction or damage. Several apoptosis pathways, including the mitochondrial pathway, the death receptor and the endoplasmic reticulum pathway, have been reported and the endogenous apoptosis pathway mediated by mitochondria composes the key part of these pathways [11,12]. In mammals, the mitochondrial pathway is the most common apoptosis mechanism of programmed cell death [13]. Furthermore, the anti-apoptotic protein Bcl-2, located in the mitochondrial, keeps the mitochondria intact and hinders the release of apoptosis-related active substances—cytochrome C and so on. However, the apoptosis-promoting protein Bax promotes the whole process. Additionally, the caspase family also plays a key role in the molecular mechanisms of inducing cell apoptosis and is ultimately involved in the execution of apoptosis, among which caspase 3, called “death protease”, makes cell apoptosis inevitable once activated [36]. It is clear that the apoptosis is closely related to mitochondrial dysfunction. In our study, we found that hypoxia can result in trophoblasts apoptosis; the mechanism should be activating mitochondrial damage and promoting caspase family gene expression and then induced apoptosis. We also observed that MSCs could attenuate apoptosis and mitochondrial damage. Our findings are consistent with Guo’s research that MSCs reduce rat granulosa cell apoptosis induced by cisplatin [26].

In our study, we observed that Mfn2 levels were increased and may have reached the maximal levels between 2 and 4 h in cells incubated with hypoxia, but started to decline and remained at their lowest levels after the 24 h point. However, when co-cultured with MSCs, Mfn2 levels appeared higher when compared to trophoblasts cultured alone. Mfn2, a conserved dynamin-like GTPases, is mainly involved in the process of mitochondrial fusion and regulates the structure and function of mitochondrial [37,38]. Mfn2 is located on the mitochondrial outer membrane and the abnormal expression may have an effect on the generation of ATP and activate the apoptosis cascade. Our findings showed that MSCs can overcome hypoxia-induced cell dysfunction and apoptosis in trophoblasts, and as this change has a positive correlation with Mfn2, we speculated that MSCs could enhance mitochondrial function and reduce apoptosis by upregulating *Mfn2* expression.

In summary, we found that bone marrow mesenchymal stem cells can attenuate mitochondria damage and apoptosis induced by hypoxia in mouse trophoblasts via regulating the expression of *Mfn2* to improve mitochondrial structure and function. However, further research should be pursued to address the mechanism of MSC upregulating of *Mfn2* and to explore the potential effect of the MSCs on gene expression and cell function.
